# Drépanocytose chez l'enfant lushois de 6 à 59 mois en phase stationnaire: épidémiologie et clinique

**DOI:** 10.11604/pamj.2014.19.71.3684

**Published:** 2014-09-24

**Authors:** Mick Ya Pongombo Shongo, Olivier Mukuku, Toni Kasole Lubala, Augustin Mulangu Mutombo, Gray Wakamb Kanteng, Winnie Sombodi Umumbu, Robert Mbuli Lukamba, Stanislas Okitotsho Wembonyama, Oscar Numbi Luboya

**Affiliations:** 1 Faculté de Médecine, Université de Lubumbashi, Lubumbashi, République Démocratique du Congo

**Keywords:** Drépanocytose, phase stationnaire, enfant, Lubumbashi, sickle cell disease, stationary phase, child, Lubumbashi

## Abstract

**Introduction:**

La drépanocytose constitue un véritable problème de santé publique avec une prévalence importante des formes majeures SS. L'objectif de notre étude est de déterminer le profil épidémiologique et clinique de la drépanocytose chez les homozygotes SS lushois âgés de 6 à 59 mois en phase stationnaire.

**Méthodes:**

Il s'agit d'une étude descriptive des aspects épidémio-cliniques des enfants drépanocytaires homozygotes SS lushois âgés de 6 à 59 mois en phase inter critique de la maladie. Les paramètres étudiés sont: l’âge, le sexe, le niveau socio-économique, l'origine ethnique, le passé médical (âge et type de la première crise et nombre de transfusion), l'appréciation de l'ictère, la classification de la splénomégalie et la recherche du déficit moteur.

**Résultats:**

La moyenne d’âge est de 39,1±16,3 mois et le sexe féminin était prédominant (53,7%). Les 3/5 de nos patients sont d'un niveau de vie bas et l'ethnie Luba représente 68,3% de cas. L’âge moyen d'apparition de la première crise se situe à 10,1±10,0 mois. La majorité a présenté sa première crise avant d'atteindre l’âge de 12 mois et plus de la moitié a présenté un syndrome pieds-main ou dactylite comme première crise. Le nombre moyen de transfusion est de 2,1 transfusions. La rate était palpable dans 73,2% des cas et l'ictère était observé dans plus de 60% de cas.

**Conclusion:**

La drépanocytose reste un véritable problème de santé publique dans notre pays. Faute de dépistage néonatal systématique, le diagnostic est souvent posé en présence d'un signe d'appel. La meilleure connaissance des différents aspects cliniques de la maladie devraient permettre de réduire la mortalité infanto-juvénile.

## Introduction

La drépanocytose constitue un véritable problème de santé publique avec une prévalence importante des formes majeures SS. Piel estime sur base des données des nations unies que 307630 bébés sont nés avec la drépanocytose en 2010 [1]. Les auteurs signalent également des grandes disparités régionales, par exemple, ils estiment moins des naissances en Inde et beaucoup plus en Afrique [2]. En Afrique, sa fréquence augmente de l'Ouest à l'Est et du Nord au Sud: le trait drépanocytaire passe de 15% au Sénégal à plus de 40% en Afrique Centrale [3]. En République Démocratique du Congo (RDC), l'OMS estime que le taux de porteur AS est de 25% et l'incidence annuelle de la forme homozygote SS autour de 15‰ naissances. Au Katanga, Tshilolo estime une prévalence attendu d'homozygotes SS de 25‰ [4].

Son expression clinique est extrêmement variable selon les groupes ethniques, mais aussi d'individu à individu. Cette hétérogénéité clinique observée serait liée à des facteurs de régulation du gène β^S^, notamment les haplotypes [5]. La RDC, pays enclavé en Afrique centrale, a la particularité de présenter une population de stock génétique en majorité encore homogène dont les sujets drépanocytaires portent essentiellement l'haplotype Bantou [6]. L'objectif de notre étude est donc de déterminer le profil épidémiologique et clinique de la drépanocytose chez les homozygotes SS lushois âgés de 6 à 59 mois en phase stationnaire.

## Méthodes

Nos recherches se sont effectuées au Centre de Prise en Charge des Drépanocytaires (CPCD) installé dans l'hôpital Sendwe dans la ville de Lubumbashi en RDC. A ce jour, ce centre suit en ambulatoire plus de 1200 drépanocytaires dont 205 homozygotes SS âgés de 6 à 59 mois. Il s'agit d'une étude descriptive des aspects épidémio-cliniques des enfants drépanocytaires homozygotes SS lushois âgés de 6 à 59 mois en phase inter critique de la maladie. La récolte de données s'est déroulée pendant 8 mois soit de juin 2012 à février 2013. La phase stationnaire ou inter critique était définie par l'absence de toute fièvre, de crise vaso-occlusive ou hémolytique.

Il s'est agi d'un tirage systématique aléatoire qui a inclus tout sujet hémoglobinopathe SS, en phase stationnaire, figurant sur la liste de drépanocytaires recensés, reçu en consultation de routine pendant la période de l’étude et âgé de 6 à 59 mois. Un consentement verbal des parents ou tuteurs était obligatoirement sollicité. Les paramètres étudiés sont: l’âge, le sexe, le niveau socio-économique, l'origine ethnique, le passé médical (âge et type de la première crise et nombre de transfusion), l'appréciation de l'ictère, la classification de la splénomégalie (selon Hackett), la recherche du déficit moteur. Pour chaque patient, les variables épidémiologiques et cliniques ont été recueillies sur une fiche d'enquête puis saisies sur ordinateur et analysées grâce au logiciel Epi-Info 2011 (version 7.0.3.8).

## Résultats

**Figure 1:**
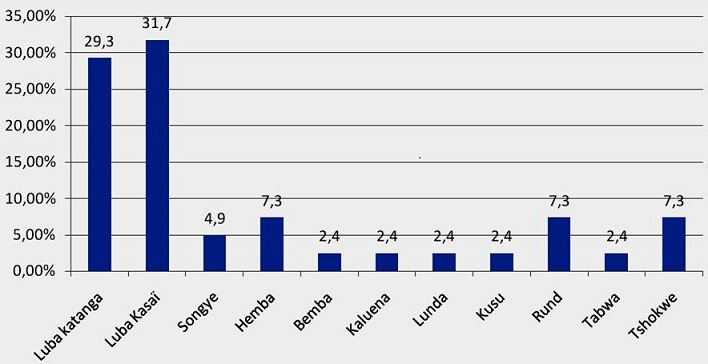
Distribution des cas selon leurs tribus d'origine

La moyenne d’âge est de 39,1 mois avec un écart-type de 16,3 (soit 3,3±1,4 ans). Le plus jeune patient était âgé de 6 mois et le plus âgé avait 59 mois. La tranche d’âge de 36 à 59 mois est la plus représentée avec près de la moitié de cas. Le sexe féminin était prédominant (53,7%) avec un sexe ratio est de 1,15 en sa faveur. Les 3/5 de nos patients sont d'un niveau de vie bas (Tableau 1). L'ethnie Luba (Luba Katanga, Luba Kasaï, Songye et Hemba) représente 68,3% de cas. La tribu Luba Kasaï représente le 1/3 de drépanocytaires homozygotes comme nous le montre la Figure 1. L’âge moyen d'apparition de la première crise dans notre série se situe à 10,1 mois avec un écart-type de 10,0. Les extrémités sont de 3 et 54 mois. La majorité de drépanocytaires SS de notre série ont présenté leur première crise avant d'atteindre l’âge de 12 mois. Plus de la moitié de drépanocytaires de notre série ont présenté un syndrome pieds-main ou dactylite comme première crise parmi lequel 95,2% avant l’âge de 12 mois. Les détails sont présentés dans le Tableau 2. Le nombre moyen de transfusion dans notre série est de 2,1 transfusions avec un écart-type de 2,30. Dans notre série, près de 4/5 des patients ont reçu au moins une transfusion. Le nombre minimal de transfusion est de 0 et le maximal est de 9. Près de 3/4 des patients de notre série avaient une rate palpable soit 73,2%. L'ictère était observé dans plus de 60% de cas. Un seul de nos patients a présenté un déficit moteur (Tableau 3).

**Tableau 1:** Aspects sociodémographiques

Paramètre	Effectif (n=41)	Pourcentage
**Tranche d’âge**		
< 12 mois	2	4,9
12-23 mois	6	14,6
24-35 mois	13	31,7
36-59 mois	20	48,8
**Sexe**		
Masculin	19	46,3
Féminin	22	53,7
**Niveau de vie**		
Bas	25	61,0
Moyen	14	34,1
Elevé	2	4,9

**Tableau 2:** Cas répartis selon l’âge de la première crise et le signe d'appel

Age 1^ère^ crise	Main-pied	Anémie	Main-pied + anémie	Autres	Total
< 12 mois	20 (95,2%)	7 (77,8%)	2 (50,0%)	2 (50,0%)	34 (82,9%)
12-23 mois	1 (4,8%)	1 (11,1%)	1 (25,0%)	2 (50,0%)	5 (12,2%)
≥24mois	0 (0,00%)	1 (11,1%)	1 (25,0%)	0 (0,0%)	2 (4,9%)
Total	21 (100%)	9 (100%)	4 (100%)	4 (100%)	41 (100%)

**Tableau 3:** Répartition des cas selon le nombre de transfusion et les signes cliniques

Paramètre	Effectif (n=41)	Pourcentage
**Nombre de transfusions**		
0	9	22,0
1	14	34,2
2	6	14,6
3	5	12,2
4	3	7,3
≥5	4	9,8
**Classification de la splénomégalie**		
0	11	26,8
1	16	39,0
2	12	29,3
3	2	4,9
**Ictère**		
Présent	26	63,4
Absent	15	36,6
**Déficit moteur**		
Présent	1	2,4
Absent	40	97,6

## Discussion

L’âge moyen de nos patients était de 3,3±1,4 ans. La tranche d’âge 36 à 59 mois était la plus représentée avec une fréquence de 48,8%. Dans notre milieu, le diagnostic est rarement posé avant l’âge de 2 ans en dehors de screening néonatal systématique [7]. La découverte précoce de la drépanocytose dans notre milieu est fonction de la précocité de signe d'appel expliquant ainsi la faible fréquence observée des drépanocytaires âgés de moins de 2 ans dans la série. Des techniques fiables de dépistage néonatal sont disponibles depuis plus de 40 ans et se perfectionnent davantage. Ces techniques permettent de dépister précocement la majorité des hémoglobinopathies notamment la drépanocytose dont il est ici question [8].

Le sexe féminin représente un peu plus de la moitié des cas (53,7%). Cette prédominance est aussi retrouvée dans l’étude de Nacoulma, qui dans sa série avait trouvé un sexe ratio de 1,5 [9]. Par contre, d'autres auteurs dont Diagne et Mabiala rapportent une légère prédominance du sexe masculin [10, 11]. Enfin, d'autres ne constatent aucune prédominance entre les deux sexes; c'est le cas de Thuilliez [12] et Dreux [13]. Ces différences seraient en rapport avec les données démographiques de chaque pays car la transmission de la drépanocytose n'est pas liée au sexe [14, 15].

Près de 70% des patients de notre série viennent des familles vivant à un niveau de vie bas. La précarité du niveau de vie entrave sur la prise en charge adéquate des drépanocytaires [16]. Les patients venant des familles vivant à un niveau de vie élevé ne représentent que 4,9% de cas dans notre série. Ceci s'expliquerait par le fait que les parents qui en ont les moyens envoient leurs enfants drépanocytaires dans les pays les mieux équipés pour une meilleure prise en charge, c'est la *migration sanitaire*. Les parents d'un niveau de vie élevé et instruits se limitent au seul enfant drépanocytaire ou prennent la précaution de faire des examens prénuptiaux, dont l’électrophorèse de l'Hémoglobine. Plus de la moitié de drépanocytaires de la série (56,1%) ont présenté une dactylite comme première crise et 82,9% ont présenté leur première crise avant d'atteindre leur premier anniversaire. Tshilolo retrouve les signes d'appel dans 80% avant l’âge de 12 mois; dans ses formes à révélation précoce, il retrouve la dactylite et/ou l'anémie [7].

Les malades viennent tous d'une population urbaine et c'est l'accessibilité de l'hôpital Jason Sendwe et la qualité des soins dispensés au CPCD qui les y font venir. Le secteur minier a longtemps contribué à l'essor économique de la ville de Lubumbashi et de la province du Katanga en général. C'est ce qui explique l'ampleur des flux migratoires vers cette province de la RDC [17]. Les malades se répartissent en 11 tribus. Tshilolo est le premier à observer que l'ancien “Empire Luba” colonial représentait le 2/3 de cas. Dans notre série, l'empire Luba représente 68,3% de cas. Tshilolo situe ce groupe de tribus majoritaires (Luba Kasaï, Luba Katanga, Songye, Hemba et Lunda), au-delà de la rivière Lualaba et au centre sud de 2 provinces du Kasaï [7]. Cependant, la distribution géographique du gène beta S ne montre pas de différence entre les différents territoires du pays [18]. Dans la série, près de 80% des patients ont reçu au moins une transfusion. De Montalembert rapporte que plus de 60% des patients homozygotes SS sont transfusés au moins une fois avant leur 18^ème^ anniversaire [19]. En RDC, 80% des sujets drépanocytaires sont transfusés avec du sang entier et le besoin annuel sanguin est estimé à 0,4 unité/patient/an [20]. A côté des situations où l'objectif est de corriger une anémie aiguë, la transfusion peut avoir pour but de diminuer le taux d'hémoglobine S pour lever l'obstruction d'un ou de plusieurs vaisseaux [21].

Au moins 70% des patients ont présenté une splénomégalie persistante. Il est bien connu que la drépanocytose présente un tableau clinique polymorphe selon les individus et les populations concernés [22]. La persistance de la splénomégalie observée dans notre étude a déjà été signalée chez des malades nigérians [23]. Il y a encore une grande controverse sur l'explication possible de cette persistance de la splénomégalie dans la clinique. Si Adekile [23, 24] soutient la thèse d'une possible réaction avec le paludisme endémique, Tshilolo n'est pas de cet avis, il penserait à une éventuelle association avec l’ α thalassémie délétionnel [7]. Nous n'avons pu faire le phénotype hémoglobinémique à la recherche d'une éventuelle association hémoglobine SS - α thalassémie. L'ictère était observé dans près de 2/3 de cas. Sangaré retrouve, dans sa série, l'ictère dans 90% des cas [25]. L'ictère est dû à l'hémolyse importante que l'on rencontre dans la drépanocytose. Il faut signaler que l'ictère apparaît consécutivement à l'anémie et classiquement après l’âge de 6 mois, âge auquel l'hémoglobine fœtale (Hb F) commence à être remplacée par l'hémoglobine S qui devient alors prédominante [26]. Nous avons observé un seul sujet avec déficit moteur hémi corporel soit 2,9%. Dans son étude à Kolwezi au Katanga, Tshilolo observe 2,7% d'accident vasculaire cérébral (AVC) dans une population quasi identique à la nôtre [7]. La drépanocytose multiplie par 220 le risque d'AVC chez l'enfant avec un risque cumulatif de 17% à 20 ans [27].

## Conclusion

La drépanocytose reste un véritable problème de santé publique dans notre pays. Faute de dépistage néonatal systématique, le diagnostic est souvent posé en présence d'un signe d'appel. La meilleure connaissance des différents aspects cliniques de la maladie devraient permettre de réduire la mortalité infanto-juvénile.
